# New record of *Trichodina unionis* (Ciliophora, Trichodinidae) from freshwater gastropods in Bangkok, Thailand

**DOI:** 10.1051/parasite/2019047

**Published:** 2019-07-30

**Authors:** Pichit Wiroonpan, Watchariya Purivirojkul

**Affiliations:** 1 Animal Systematics and Ecology Speciality Research Unit, Department of Zoology, Faculty of Science, Kasetsart University, Bang Khen Campus Bangkok 10900 Thailand

**Keywords:** Ciliate protozoa, Freshwater snails, Molecular analysis, Phylogenetic tree, Trichodinid

## Abstract

Trichodinids, which are ciliate protists, are causative agents of an aquatic animal disease, trichodiniasis, especially among both captive and wild fish. This disease can adversely affect aquaculture and have economic impacts. The objectives of this study were to evaluate the prevalence and mean intensity of *Trichodina unionis* infection, describe qualitative and quantitative morphological characters, and perform a molecular phylogenetic analysis. The gastropod samples were randomly collected by hand-picking and a hand net. *Trichodina unionis* was collected by the crushing method under a stereomicroscope. Among all 4977 examined gastropods, 55 individuals of two gastropod species, *Gyraulus siamensis* and *Physella acuta*, were found to be infected by *T. unionis*, with overall prevalence and mean intensity of infection of 1.11% and 16.65, respectively. The characteristics of the denticles indicated *T. unionis* as having moderately wide blades and moderately curved blade margins, with distinctive bend angles near the distal end. The quantitative characters showed some variations, which could be due to food availability. Molecular phylogenetic analysis conducted with 18S rRNA provided a monophyletic tree of our specimens and previously identified *T. unionis*, confirming species identification. This study represents the first record of *T. unionis* in Thailand.

## Abstract (fr)

Les Trichodinidae, qui sont des protistes ciliés, sont des agents responsables d’une maladie des animaux aquatiques, la trichodiniase, en particulier chez les poissons captifs et sauvages. Cette maladie peut avoir des effets négatifs sur l’aquaculture et un impact économique. Les objectifs de cette étude étaient l’évaluation de la prévalence et de l’intensité moyenne de l’infection par *Trichodina unionis*, la description des caractères morphologiques qualitatifs et quantitatifs et une analyse phylogénétique moléculaire. Les échantillons de gastéropodes ont été prélevés au hasard à l’aide d’une récolte manuelle et d’un filet. *Trichodina unionis* a été collecté par la méthode de concassage sous stéréomicroscope. Parmi les 4977 gastropodes examinés, 55 individus appartenant à deux espèces de gastéropodes, *Gyraulus siamensis* et *Physella acuta*, ont été trouvés infectés par *T. unionis*, avec une prévalence globale et une intensité d’infection moyenne de 1,11 % et 16,65, respectivement. Les caractéristiques des denticules indiquaient que *T. unionis* avait des lames moyennement larges et des bords de lame moyennement incurvés avec des angles de courbure distincts près de l’extrémité distale. Les caractères quantitatifs présentaient quelques variations, qui pourraient être dues à la disponibilité de la nourriture. L’analyse phylogénétique moléculaire réalisée avec l’ARNr 18S a fourni un arbre monophylétique de nos spécimens et de *T. unionis* précédemment identifiés, confirmant l’identification de l’espèce. Cette étude représente le premier signalement de *T. unionis* en Thaïlande.

## Introduction

*Trichodina*, a genus of ciliate protists, belongs to the family Trichodinidae and is well known as the causative agent of trichodiniasis in numerous aquatic animals, especially both cultured and wild fish [23–25, 38]. Sometimes, poor health in cultured fish is a clinical sign caused by this protist, leading to negative impacts on the cultured fish as well as economic impacts. *Trichodina* can serve as a facultative ectoparasite and can proliferate and invade hosts during unsuitable conditions in environments, such as poor water quality and food deficiency [15, 17]. In general, *Trichodina* are usually found on the skin and gills of marine, blackish and freshwater fish. Some species of *Trichodina* have also been reported in freshwater mussels, freshwater gastropods and tadpoles [4, 5, 8, 12, 13, 16]. In Thailand, *Trichodina* has been rarely reported, with a few studies showing the occurrence of this genus on the basis of morphological characteristics conducted in Nakhon Si Thammarat, Pattani and Trang provinces [21, 29, 33]. Only Worananthakij and Maneepitaksanti [39] addressed *Trichodina* at the species level, with three species found to be infecting red tilapia from Pathum Thani province, with identification based only on morphological characters.

The taxonomic history of *Trichodina unionis* has never been reported in Thailand. *Trichodina unionis* was first described in Schwabach, Germany, in 1955 by Hampl [13]. It was found to infect the gills of freshwater unionid mussels, i.e., *Unio crassus batavus* and *Anodonta cygnea*. Six years later, several freshwater bodies in Poland were found to be infected with *T. unionis* in the same host as in a previous report [30]. In 1965, Fenchel [12] also found this trichodinid in *A. cygnea* from Copenhagen, Denmark. Recently, *T. unionis* was found to infect an aquatic pulmonate snail, *Stagnicola* sp., from Canada by Irwin et al. [16] in 2017.

To better understand the new record of *T. unionis* in Thailand, the infection pattern in terms of prevalence and mean intensity of *T. unionis* in natural freshwater gastropods was investigated. Additionally, we describe the basic morphology of this trichodinid species, including both qualitative and quantitative characters. Biological molecular analysis to confirm the morphologically identified *T. unionis* and an investigation of the relationships between *T. unionis* and other trichodinid species were also conducted in this study.

## Materials and methods

### Ethics statement

The present study was approved by the ethics committee of Kasetsart University (approval No. ACKU61-SCI-033) for rearing the collected gastropod specimens during the observation of *T. unionis* infection.

### Specimen collection and infection investigation

**Figure 1:**
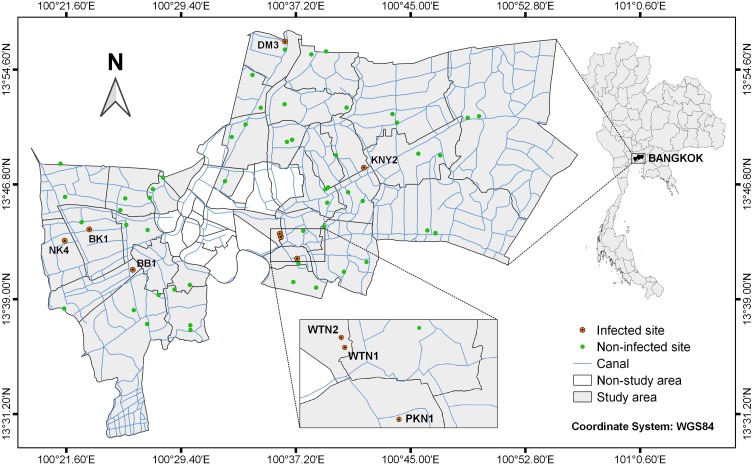
Map of study area in Bangkok, Thailand. Abbreviated names of the infected sites are as follows: BB1; Bang Bon 1; BK1: Bang Khae 1; DM3: Don Mueang 3; KNY2: Khan Na Yao 2; NK4: Nong Khaem 4; PKN1: Pra Khanong 1; WTN1: Wattana 1; WTN2: Wattana 2.

A total of 59 localities in 32 districts of Bangkok were selected as sampling sites (Fig. 1). The geographical coordinates of each sampling site were recorded by global positioning system (GPS) and were then used to construct the study area map via free QGIS software (version 3.6.1) using the WGS84 coordinate system. Several species of freshwater gastropods were randomly collected from the water bodies during August–December 2018. The gastropod specimens were collected for approximately 10–20 min at each locality [28] by hand-picking and with a hand net. The collected gastropods were morphologically identified following Brandt [6] and Upatham et al. [35] and temporarily reared in 2 L plastic boxes. *Trichodina unionis* was removed from the collected gastropods with the crushing method [7]. Briefly, the gastropods were frozen for anesthetization, and their shells were broken. The gastropod bodies were individually pressed between two Petri dishes, and *T. unionis* was observed under a stereomicroscope at high magnification. The prevalence of *T. unionis* infection was calculated by dividing the number of infected gastropods by the number of examined gastropods, and then multiplying this value by 100. The mean intensity of infection was calculated by dividing the number of *T. unionis* individuals by the number of infected gastropods. The trichodinid specimens were immediately preserved in a 1.5 mL microcentrifuge tube that contained absolute ethanol for molecular biological study.

### Morphological study

**Figure 2:**
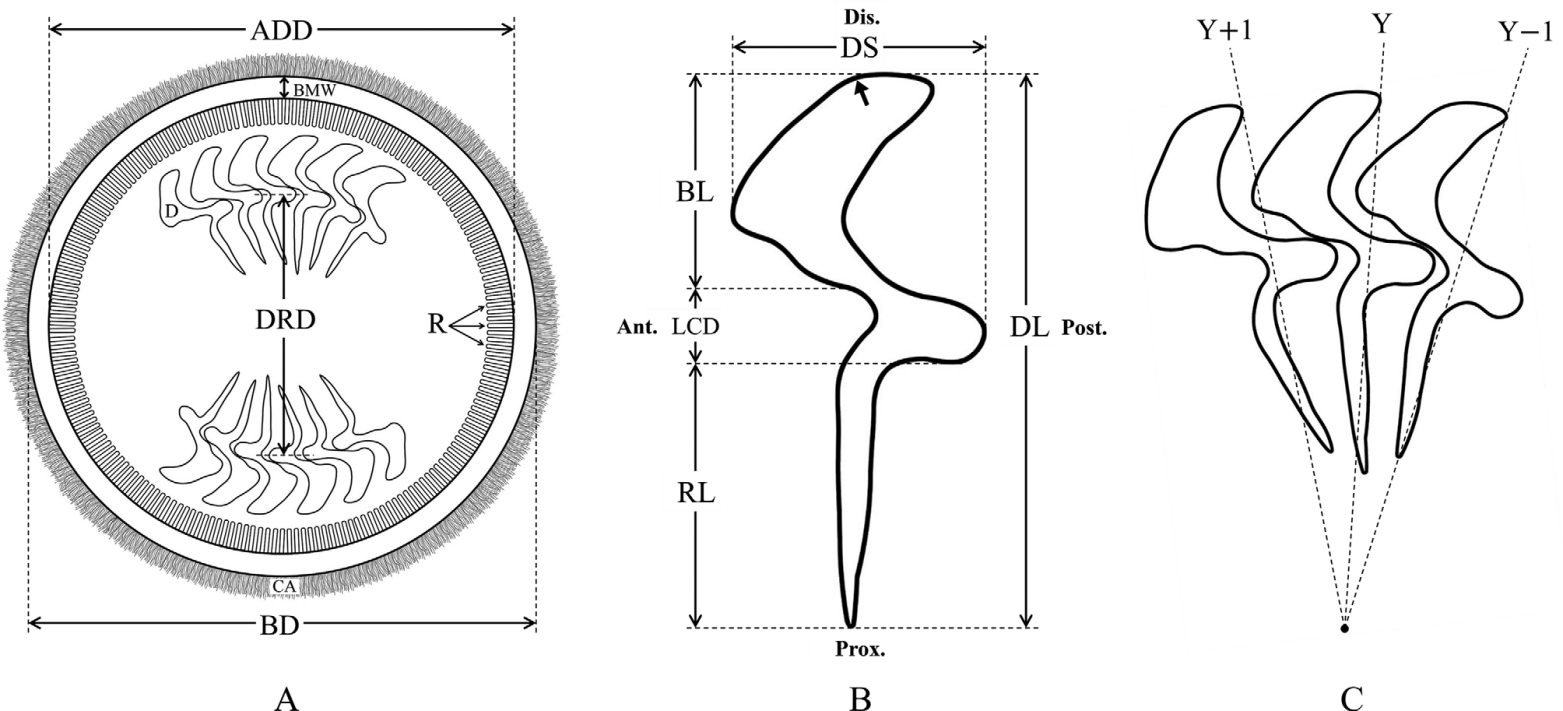
Measured and counted morphological characters of *Trichodina unionis*. (A) Morphological characters of the aboral side, (B) morphological characters of a denticle with the bold arrow indicating the distinctive bend angle on the blade margin, and (C) denticle arrangement. Abbreviations are as follows: Ant.: anterior side; ADD: adhesive disc diameter; BD: Body diameter; BMW: Border membrane width; CA: Cilia area; D: denticle; Dis.: Distal end; DRD: Denticular ring diameter, BL: Blade length; DL: Denticle length; DS: Denticle span; LCD: Length of central part of denticles; Post.: Posterior side; Prox.: Proximal end; R: Radial pin; RL: Ray length.

The living specimens of *T. unionis* were immediately collected from the host tissue, after which the specimens were placed on a glass slide, followed by air drying. Two percent silver nitrate was utilized to impregnate the dried specimens following Klein’s method [18]. Then, the silver-impregnated specimens were photographed with an Olympus BX51 compound light microscope with a DP70 camera (Olympus Corporation, Japan). Each individual photographed trichodinid was measured for morphological characters via the free software ImageJ [1, 32]. All morphological characteristics were described following Lom [22] and Van As and Basson [38], including body diameter, adhesive disc diameter, denticular ring diameter, border membrane width, denticle length, blade length, central part length of denticle, ray length and denticle span (Fig. 2). Most of the morphological characters were represented as the arithmetic mean ± standard deviation followed by minimum and maximum (range) values in parentheses, except the number of denticles, which was described as the mode with minimum and maximum values in parentheses.

### DNA extraction, PCR amplification, purification and sequencing

Approximately 8–10 individuals of the preserved *T. unionis* were washed twice with ultrapure water in a 1.5 mL microcentrifuge tube and centrifuged at 10,000 × *g* for 1 min. Genomic DNA of the trichodinids was extracted using a GF-1 Tissue DNA Extraction Kit (Vivantis, Malaysia), following the manufacturer’s instructions. The extracted samples were kept at −20 °C to maintain the integrity of the DNA. The 18S rRNA region of *T. unionis* was amplified using polymerase chain reaction (PCR) with the ciliate-specific primers following Dopheideet al. [9], i.e., 384F: YTB GAT GGT AGT GTA TTG GA for the forward primer and 1147R: GAC GGT ATC TRA TCG TCT TT for the reverse primer. These primers were expected to give approximately 750 base pairs of the amplified product. PCR amplification was conducted in a total reaction volume of 50 μL, consisting of 1X PCR buffer, 2 mM MgCl_2_, 0.2 μM forward primer, 0.2 μM reverse primer, 100 μM dNTP mixture, 2 units/μL Taq polymerase, and 2 μL DNA template. PCR conditions were 5 min at 94 °C for predenaturation, 30 cycles of 45 s at 94 °C, 60 s at 55 °C, and 90 s at 72 °C for denaturation, annealing and extension, performed in a thermal cycler (Mastercycler Pro, Eppendorf, Germany). Finally, a final extension step was conducted for 7 min at 72 °C. Gel electrophoresis was used to observe the presence of PCR products of the correct size in 1.5% agarose gel stained with SYBR safe (Invitrogen) at 50 V for 60 min. The amplified DNA samples were purified and sequenced by Macogen, Korea, using the same forward and reverse primers that were used for the PCR.

### Molecular identification and phylogenetic analysis

The presence of the expected PCR products in the trichodinid DNA sequence data, consisting of 3PW1 (from *Gyraulus siamensis*), 29BB1 (from *Physella acuta*) and 99BB1 (from *G. siamensis*), was confirmed using the standard nucleotide basic local alignment search tool (BLAST) with megablasts from the NCBI database. Before confirmation, the sequence data obtained with the forward and reverse primers for each DNA sample were assembled as a contig (a set of contiguous sequences) using the CAP3 sequence assembly program [14], and then the sequence portions near the sequencing primer sites of each contig were trimmed for accurate construction of the phylogenetic tree. Thereafter, the three current sequence datasets and the 20 related sequence datasets that were acquired from the NCBI database (Table 1) were aligned via ClustalW in MEGA7 software [20]. All aligned sequences were used to construct the phylogenetic tree using the maximum likelihood statistical method based on the General Time Reversible (GTR) model [26] with 10,000 bootstrap tests in MEGA7 software [20]. *Urceolaria urechi* and *U. korschelti* were used as an outgroup. Furthermore, the p-distance method of pairwise distance analysis was also used to estimate the evolutionary divergence of the trichodinids via MEGA7 software. The DNA sequences of 18S rRNA region of current trichodinids were submitted to GenBank, including MN082435 (3PW1), MN082436 (29BB1) and MN082437 (99BB1) (Table 1).

**Table 1:** Sequence data for the 18S rRNA region of the current trichodinids (bold taxa) and trichodinids from the NCBI database used for molecular phylogenetic analysis.

Species	Accession number
**3PW1 *Trichodina unionis***	MN082435
**29BB1 *Trichodina unionis***	MN082436
**99BB1 *Trichodina unionis***	MN082437
*Trichodina reticulata*	AY741784.1
*Trichodina heterodentata*	AY788099.1
*Trichodina ruditapicis*	FJ499385.1
*Trichodina sinonovaculae*	FJ499386.1
*Trichodina meretricis*	FJ499387.1
*Trichodina unionis*	KY596041.1
*Trichodina domerguei*	KY596035.1
*Trichodina tenuidens*	KY596040.1
*Trichodina pectenis*	JQ663868.2
*Trichodina truttae*	LC186029.1
*Trichodina centrostrigata*	KP295473.1
*Trichodina nobilis*	AY102172.1
*Trichodina pseudoheterodentata*	KT804995.1
*Trichodina hyperparasitis*	KX904933.1
*Trichodina acuta*	KX904932.1
*Trichodina sinipercae*	EF599288.1
*Trichodina modesta*	GU906245.1
*Trichodina hypsilepis*	EF524274.1
*Urceolaria urechi*	FJ499388.1
*Urceolaria korschelti*	JQ663870.1

## Results

### Trichodinid infection of the gastropods

**Figure 3:**
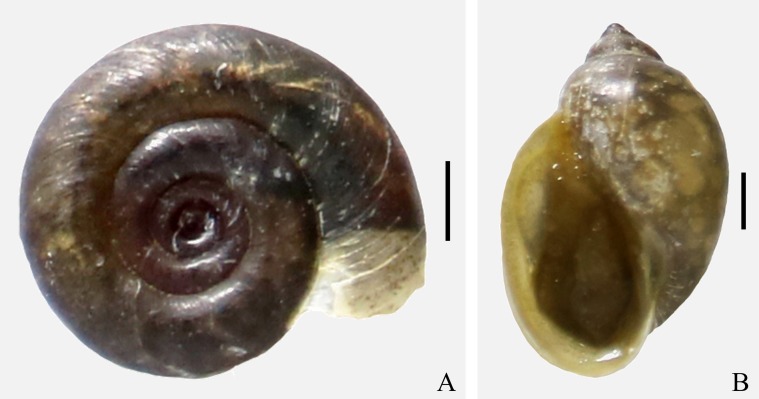
Two freshwater gastropods infected by *Trichodina unionis*. (A) *Gyraulus siamensis*, (B) *Physella acuta*. Scale bars: 1 mm.

All 4977 individuals of the collected freshwater gastropods were examined for trichodinid infection, and 55 of the examined gastropods (belonging to two species, i.e., *Gyraulus siamensis* and *Physella acuta* (Fig. 3) were found to be infected with *T. unionis*. The overall prevalence and mean intensity of trichodinid infection were 1.11% and 16.65, respectively. The mean intensity of infection was higher in *G. siamensis* (21.53) in comparison to *P. acuta* (7.42). No trichodinids were found in 16 species of 8 families of freshwater gastropods (Table 2). Eight of 59 localities were found to have freshwater gastropods infected with *T. unionis*, which were Bang Bon 1 (13°40′59.9″N 100°26′06.6″E), Bang Khae 1 (13°43′44.5″N 100°23′09.4″E), Don Mueang 3 (13°56′30.3″N 100°36′27.5″E), Khan Na Yao 2 (13°47′56.0″N 100°41′49.1″E), Nong Khaem 4 (13°42′58.3″N 100°21′28.6″E), Pra Khanong 1 (13°41′45.3″N 100°37′17.0″E), Wattana 1 (13°43′14.1″N 100°36′10.1″E) and Wattana 2 (13°43′26.5″N 100°36′05.9″E) (Fig. 1).

**Table 2:** Species and number of examined gastropods collected from Bangkok with their infective data.

Examined snails	Number of examined snails	Number of infected snails	Number of parasites	Prevalence (%)	Mean intensity
Ampullariidae					
*Pomacea canaliculata*	283	–	–	–	–
Bithyniidae					
*Bithynia siamensis siamensis*	648	–	–	–	–
Buccinidae					
*Clea helena*	217	–	–	–	–
Lymnaeidae					
*Lymnaea auricularia rubiginosa*	362	–	–	–	–
*Lymnaea viridis*	6	–	–	–	–
Pachychilidae					
*Adamietta housei*	78	–	–	–	–
Physidae					
*Physella acuta*	290	19	141	6.55	7.42
Planorbidae					
*Gyraulus siamensis*	139	36	775	25.90	21.53
*Indoplanorbis exustus*	180	–	–	–	–
Thiaridae					
*Melanoides tuberculata*	58	–	–	–	–
*Sermyla riqueti*	25	–	–	–	–
*Tarebia granifera*	335	–	–	–	–
*Thiara scabra*	22	–	–	–	–
Viviparidae					
*Filopaludina cambodiensis*	2	–	–	–	–
*Filopaludina martensi*	997	–	–	–	–
*Filopaludina polygramma*	1284	–	–	–	–
*Filopaludina speciosa*	42	–	–	–	–
*Idiopoma umbilicata*	9	–	–	–	–
Overall	4977	55	916	1.11	16.65

### Morphological description

**Figure 4:**
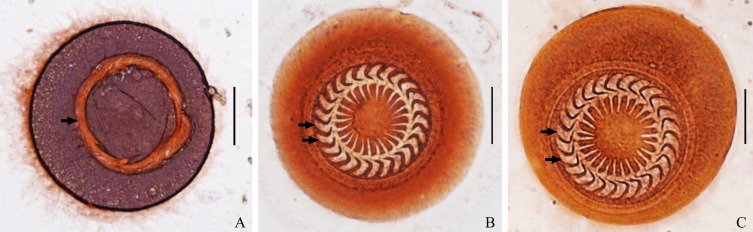
Morphological photomicrographs of the dry silver-impregnated *Trichodina unionis* from snail *Physella acuta* (A and B) and snail *Gyraulus siamensis* (C). (A) Complete-turned ciliature on the oral side (arrow), (B and C) circular arrangement of the denticles within the adhesive disc on the aboral side (arrows point to the bend angle on the blade margin of the denticle). Scale bars: 20 μm.

In all, 53 individuals of *T. unionis* from two freshwater gastropod hosts were investigated for their morphological characteristics. These trichodinids were medium-sized and showed a disc-shaped body. The oral side of the trichodinids was characterized as a convex surface with a complete spiral turn of ciliature (Fig. 4A), and the aboral side showed a slightly concave surface of the adhesive disc. However, no granules within the central part of the adhesive disc of the silver-impregnated specimens were observed. Many denticles on the aboral side were distinctly represented as having a circular arrangement on the adhesive disc (Fig. 4). Each denticle was sickle shaped. The denticles can be characterized as having a moderately wide blade with a rounded end point near the border membrane. Remarkably, the blade margin near the distal end was moderately falcate with a distinctive curved angle (Figs. 2B and 4). Mostly, the blade fills the space between the *Y* and *Y* − 1 axes and some space that is to the left of the *Y* axis. Nevertheless, a small space to the left side of the *Y* + 1 axis was also filled by a few blades (Fig. 2C). Rays were long and straight with a slight difference in length and tapered to the proximal end point near the central part of the adhesive disc. The central part of the denticles had a triangular shape and a well-developed apophysis in the posterior direction; in contrast, the anterior direction had a concave area that fits and supports the apophysis of the adjacent denticle.

Several measured and counted morphological characters of the trichodinid were also described (Table 3) as follows: 50.5 ± 4.1 (42.5–57.9) body diameter, 44.1 ± 4.0 (36.2−51.6) diameter of the adhesive disc, 26.7 ± 2.5 (20.7−31.6) denticular ring diameter, 3.3 ± 0.3 (2.6−3.9) border membrane width, 25.0 (22−30) denticle number, 12.4 ± 1.1 (10.0−14.1) denticle length, 5.8 ± 0.5 (4.7−6.6) blade length, 2.0 ± 0.3 (1.4−2.7) length of central part of denticles, 5.3 ± 0.6 (3.5−6.2) ray length, and 7.3 ± 0.7 (5.5−8.6) denticle span (denticle width). However, the number of radial pins was not recorded because of the indistinctness of this character in the silver-impregnated samples.

**Table 3:** Measured and counted characters of *Trichodina unionis* between the present study and previous studies.

Author	Present study	Hampl [13]	Fenchel [12]	Irwin et al. [16]
Study area	Bangkok, Thailand	Schwabach, Germany	Copenhagen, Denmark	British Columbia, Canada
Host	Freshwater gastropods	Freshwater mussels	Freshwater mussels	Pulmonate freshwater gastropods
Number of specimens	53	–	–	3
Body diameter (μm)	50.5 ± 4.1	–	70.0	56.0 ± 6.0
	(42.5–57.9)	(60.0–90.0)	–	(50.0–62.0)
Adhesive disc diameter (μm)	44.1 ± 4.0	–	–	46.0 ± 6.2
	(36.2–51.6)	(58.0–62.0)	–	(41.0–53.0)
Denticular ring diameter (μm)	26.7 ± 2.5	–	26.0	26.0 ± 2.6
	(20.7–31.6)	(29.0–31.0)	–	(24.0–29.0)
Border membrane width (μm)	3.3 ± 0.3	–	–	4.0 ± 1.4
	(2.6–3.9)	–	–	(3.0–5.0)
Denticle number	25.0	–	26.0	25.0
	(22–30)	(26–33)	(22–28)	(22–28)
Denticle length (μm)	12.4 ± 1.1	–	–	–
	(10.0–14.1)	–	–	–
Blade length (μm)	5.8 ± 0.5	7.0	–	7.0 ± 1.0
	(4.7–6.6)	–	–	(6.0–8.0)
Central part length (μm)	2.0 ± 0.3	–	–	–
	(1.4–2.7)	–	–	–
Ray length (μm)	5.3 ± 0.6	9.0	–	7.3 ± 1.2
	(3.5–6.2)	–	–	(6.0–8.0)
Denticle span (μm)	7.3 ± 0.7	–	–	–
	(5.5–8.6)	–	–	–

### Molecular analysis

**Figure 5:**
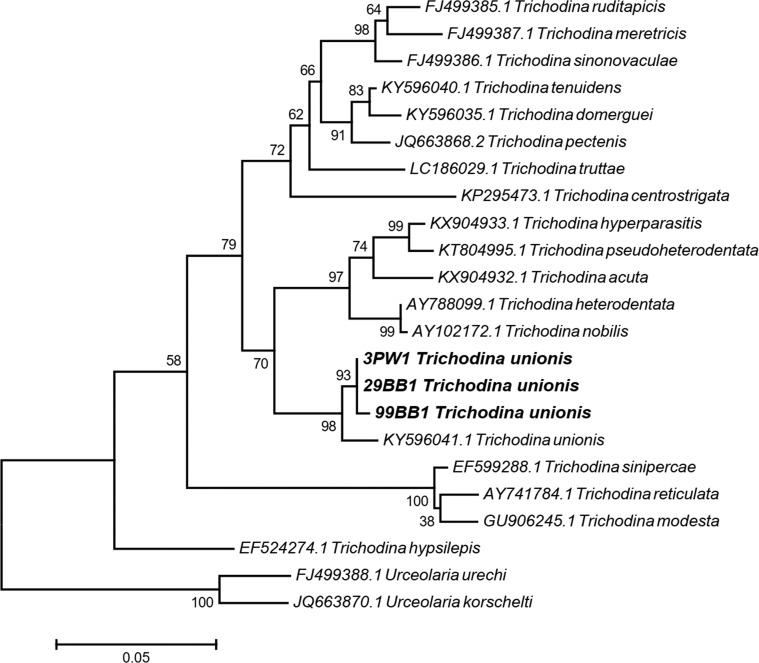
The rooted phylogenetic tree of the trichodinids from the partial 18S rRNA region using the maximum likelihood method. Numbers at each node represent maximum likelihood bootstrap values from 10,000 replicates. The bold taxa refer to trichodinids in this study. The scale bar was drawn as branch lengths measured in the number of substitutions per site of nucleotides.

Phylogenetic analysis of the trichodinids according to 526 base pairs of the 18S rRNA region of each sample was performed using the statistical method of maximum likelihood with 10,000 bootstrap tests for species confirmation between the current *T. unionis*, which was initially identified based on morphological characters, and other species of *Trichodina* as well as the determination of their relationships. The phylogram of the relationships among *Trichodina* was shown as a monophyletic tree, with *Urceolaria urechi* and *U. korschelti* being considered as an outgroup. Interestingly, three present trichodinid samples, 3PW1 (MN082435) from *G. siamensis*, 29BB1 (MN082436) from *P. acuta*, and 99BB1 (MN082437) from *G. siamensis*, and *T. unionis* (KY596041) were closely grouped together as a monophyletic group with a high probability according to the bootstrap test value (Fig. 5). Additionally, the pairwise distance analysis provided small values of p-distance both within the current *T. unionis* samples (0.0%–0.4%) and between the current *T. unionis* and previous *T. unionis* (KY596041.1) (1.5%–1.9%) (Table 4). However, the phylogram indicated a non-monophyletic tree for *T. unionis* and *T. heterodentata*; *T. heterodentata* is commonly found in Thailand.

**Table 4:** Pairwise analysis of distance estimation among sequence data of the trichodinids derived from the 18S rRNA region using the p-distance method. The bold taxa referred to the present trichodinids.

	1	2	3	4	5	6	7	8	9	10	11	12	13	14	15	16	17	18	19	20	21	22
1. **3PW1 *T. unionis***																						
2. **29BB1 *T. unionis***	0.000																					
3. **99BB1 *T. unionis***	0.004	0.004																				
4. *T. reticulata*	0.118	0.118	0.122																			
5. *T. heterodentata*	0.055	0.055	0.059	0.125																		
6. *T. ruditapicis*	0.074	0.074	0.078	0.116	0.068																	
7. *T. sinonovaculae*	0.072	0.072	0.076	0.122	0.067	0.021																
8. *T. meretricis*	0.074	0.074	0.078	0.125	0.067	0.027	0.029															
9. *T. unionis*	0.015	0.015	0.019	0.120	0.059	0.080	0.074	0.072														
10. *T. domerguei*	0.057	0.057	0.061	0.108	0.070	0.049	0.048	0.046	0.067													
11. *T. tenuidens*	0.061	0.061	0.065	0.112	0.067	0.042	0.036	0.038	0.063	0.011												
12. *T. pectenis*	0.067	0.067	0.070	0.122	0.074	0.051	0.046	0.046	0.067	0.023	0.019											
13. *T. truttae*	0.070	0.070	0.074	0.124	0.074	0.063	0.055	0.057	0.072	0.053	0.049	0.049										
14. *T. centrostrigata*	0.078	0.078	0.082	0.118	0.091	0.078	0.080	0.086	0.084	0.065	0.067	0.072	0.084									
15. *T. nobilis*	0.057	0.057	0.061	0.127	0.002	0.070	0.068	0.068	0.061	0.072	0.068	0.076	0.076	0.093								
16. *T. pseudoheterodentata*	0.061	0.061	0.065	0.133	0.038	0.082	0.080	0.076	0.061	0.080	0.074	0.082	0.082	0.101	0.040							
17. *T. hyperparasitis*	0.063	0.063	0.067	0.135	0.034	0.080	0.078	0.074	0.063	0.078	0.072	0.080	0.080	0.099	0.036	0.011						
18. *T. acuta*	0.065	0.065	0.068	0.131	0.040	0.080	0.078	0.078	0.061	0.082	0.076	0.084	0.070	0.097	0.042	0.034	0.032					
19. *T. sinipercae*	0.114	0.114	0.118	0.017	0.125	0.116	0.122	0.125	0.118	0.108	0.112	0.120	0.124	0.118	0.127	0.125	0.131	0.129				
20. *T. modesta*	0.120	0.120	0.124	0.023	0.118	0.116	0.120	0.124	0.120	0.108	0.112	0.118	0.124	0.118	0.120	0.122	0.124	0.120	0.017			
21. *T. hypsilepis*	0.097	0.097	0.101	0.120	0.084	0.103	0.103	0.101	0.105	0.093	0.091	0.093	0.110	0.108	0.086	0.089	0.089	0.087	0.114	0.110		
22. *Urceolaria urechi*	0.156	0.156	0.160	0.152	0.146	0.146	0.144	0.148	0.156	0.139	0.141	0.148	0.156	0.156	0.148	0.163	0.158	0.154	0.154	0.154	0.137	
23. *U. korschelti*	0.156	0.156	0.160	0.152	0.146	0.148	0.146	0.154	0.158	0.144	0.146	0.154	0.154	0.156	0.148	0.156	0.154	0.152	0.144	0.146	0.135	0.042

## Discussion

A lower prevalence of trichodinid infection in gastropod hosts was found in this study in comparison with previous studies. Fenchel [12] reported almost 100% *T. unionis* infection in the freshwater mussel *Anodonta cygnea*. In general, the freshwater mussel population has a clumped distribution and has a sessile lifestyle in the nearby area [3, 31], leading to easy infection among individuals in the area in comparison to freshwater gastropods, which can be randomly distributed. However, a high prevalence (44%) of *T. unionis* infection in a pulmonate freshwater gastropod was also reported by Irwin et al. [16], which may have resulted from the low diversity of the examined snails. This study showed that the mean intensity of *T. unionis* infection in the gastropods was slightly higher than that reported in freshwater mussels (about ten ciliates per mussel) [12]. This may be due to the difference in water flow throughout the gills or body cavity of the freshwater mussels and freshwater gastropods. Commonly, mussels use their siphon muscles together with the cilia on their gills to adjust the volume of water flowing between the inside and outside of their body cavity, while only the cilia on the gills are used to adjust the water flow in gastropods [36]. High water pressure within the body cavity due to siphon muscle activity could withdraw and eliminate the gill-attached trichodinids out of the mussels, leading to a low number of trichodinids in the mussels.

In terms of the host specificity of the trichodinids, the ability of *T. unionis* to inhabit different families of snail hosts was found in several previous studies [4, 5, 12, 13, 16, 30]. This study also found *T. unionis* in the two gastropods that have never been reported as trichodinid hosts, which may indicate low host specificity due to wide distribution in various hosts. Other trichodinids, such as *T. heterodentata*, are usually reported in various fish and some tadpoles. Previous studies [2, 8, 10, 19, 24] revealed that *T. heterodentata* was found in at least 50 species from 16 families of fish and 4 species of tadpoles, indicating the low host specificity of *T. heterodentata* [8, 27]. However, Martins et al. [25] noted that the host specificity of trichodinids could be assumed to depend on environmental quality.

On the basis of the overall qualitative characteristics of the *Trichodina unionis* morphology, the current specimens do not look different from those in the original description by Hampl [13] in 1955, and the recent study in 2017 by Irwin et al. [16]. Nevertheless, a slight difference in the blade margin was observed. Fenchel [12] showed a slight curvature of the blade margin, while a moderately curved blade margin was observed in this study. However, the distinctive bend angle on the blade margin was similarly observed. Several quantitative characters of the trichodinid showed some variation in the morphological characters. Only one counted character, number of denticles, seemed to be certainly similar to that found in the original description [13] and two previous studies [12, 16]. Normally, the denticle number in members of the same species is not necessarily absolutely equal. Changes in denticle number can occur in new generations of trichodinids, and it is possible that denticle number depends on food availability [27]. The diameters of the adhesive disc and denticular ring in this study were almost equal to those found in a pulmonate freshwater snail described in the recent study [16], but smaller than those found in freshwater mussels reported in the original description [13]. The body diameter, border membrane width, blade length and ray length in the current trichodinid were smaller than those found in the original description [13] and two previous studies [12, 16]. Overall, smaller trichodinids were found in this study. Nevertheless, Ogut and Altuntas [27] noted that morphological changes in trichodinids could be caused mainly by food availability in the environment across different localities or different hosts. For example, a decrease in denticle size (blade length and ray length) in trichodinids living in food-scarce environments may encourage more efficient food filtering. Moreover, specimen preparation by the straining method can sometimes lead to slight differences in morphological measurements.

In comparison to other trichodinids, the denticle morphology of *T. unionis* looks somewhat similar to that of *T. heterodentata*, being sickle shaped with wide blades and tapered rays. In contrast, moderately wide blades and moderately curved blade margins were observed in *T. unionis,* while *T. heterodentata* showed a strongly wide blade and a strongly curved blade margin, as described in previous studies [8, 11, 25, 34, 37, 38]. Additionally, *T. unionis* clearly showed a bend angle on the blade margin, but this characteristic was not found in *T. heterodentata*.

Due to the similarity in some organelles, especially denticles, and the variation in quantitative characters of the trichodinids, it is difficult to identify species on the basis of classical techniques. Therefore, molecular biological techniques were used to achieve the accurate identification and confirmation of trichodinid species. The phylogenetic tree and pairwise distance analysis indicated the close relationship between *T. unionis* found in this study and *T. unionis* in the recent study by Irwin et al. [16]. Thus, the current trichodinid samples were correctly confirmed as *T. unionis*. Moreover, the current trichodinid was similarly discovered in freshwater gastropods, as found in a recent study. Therefore, it is possible that the host-preference adaptation of the trichodinid for inhabiting the same host group leads to a close evolutionary relationship. In the relationship with *T. heterodentata* that is commonly found in Thailand [39], the phylogram distinctly showed the different groups of two clades (the one made up of *T. unionis* and the sister clade containing *T. heterodentata*). *T. unionis* and *T. heterodentata* are usually found in freshwater molluscs and freshwater fish, respectively, which may have caused the differences in adaptation and host preference of the trichodinids between these two clades.

In conclusion, the present study reports the presence of the ciliate protozoan *T. unionis* for the first time in Thailand, providing additional information about the geographical distribution of *T. unionis*. Two gastropod species, *Gyraulus siamensis* and *Physella acuta*, were recorded as the new hosts, indicating the low host specificity of this trichodinid. This study showed a low prevalence and mean intensity of infection. Nevertheless, infection by this trichodinid species of other aquatic animals, such as fish, tadpoles, etc., in the same water bodies should be evaluated to better understand host specificity. The pathogenicity of *T. unionis* in other gastropods or molluscs should also be assessed to prevent trichodiniasis. Biotic and abiotic factors that may importantly relate to trichodinid infection should be further evaluated.
